# 腺苷/A2AR信号通路阻断在肿瘤治疗中的应用

**DOI:** 10.3779/j.issn.1009-3419.2022.102.24

**Published:** 2022-07-20

**Authors:** 佳 刘, 岳泉 石, 潇衍 刘, 东明 张, 瑜 白, 燕 徐, 孟昭 王

**Affiliations:** 1 100730 北京，中国医学科学院，北京协和医学院，北京协和医院呼吸与危重症医学科 Department of Respiratory and Critical Care Medicine, Peking Union Medical College Hospital, Chinese Academy of Medical Sciences and Peking Union Medical College, Beijing 100730, China; 2 201203 上海，迪哲医药有限公司 Dizal Pharmaceutical Co., Ltd., Shanghai 201203, China

**Keywords:** 肿瘤微环境, 腺苷, 腺苷受体, A2A受体拮抗剂, Tumor microenvironment, Adenosine, Adenosine receptor, A2AR antagonist

## Abstract

腺苷是一种在肿瘤微环境中高水平产生的代谢产物，主要通过在免疫细胞上表达的腺苷A2A受体（adenosine A2A receptor, A2AR）有效地抑制炎症组织中的免疫反应，抑制抗肿瘤免疫应答。因此阻断腺苷信号通路是促进抗肿瘤免疫的重要方向。在这篇文章中，我们将简介腺苷信号通路，总结腺苷/A2AR在抑制肿瘤免疫中的作用，并介绍腺苷/A2AR通路阻断在抗肿瘤治疗中的研究现状。临床前研究数据已揭示了抑制A2AR治疗肿瘤的潜在效力，A2AR拮抗剂从基础研究向临床应用的转化也正在进行当中。临床试验的初步结果显示，A2AR拮抗剂在肿瘤患者中安全性良好，无论单药还是联合其他疗法使用均有一定的疗效。未来仍需进一步探索如何选取生物标志物对潜在获益人群进行筛选，以及如何联用A2AR拮抗剂和其他治疗方案以实现最优的抗肿瘤策略。

## Abstract (en)

Adenosine is a metabolite produced abundantly in the tumor microenvironment, dampening immune response in inflamed tissues via adenosine A2A receptor (A2AR) which is widely expressed on immune cells, inhibiting anti-tumor immune response accordingly. Therefore, blocking adenosine signaling pathway is of potential to promote anti-tumor immunity. This review briefly introduces adenosine signaling pathway, describes its role in regulating tumor immunity and highlights A2AR blockade in cancer therapy. Prospective anti-tumor activity of adenosine/A2AR inhibition has been revealed by preclinical data, and a number of clinical trials of A2AR antagonists are under way. Primary results from clinical trials suggest that A2AR antagonists are well tolerated in cancer patients and are effective both as monotherapy and in combination with other therapies. In the future, finding predictive biomarkers are critical to identify patients most likely to benefit from adenosine pathway blockade, and further researches are needed to rationally combine A2AR antagonists with other anti-tumor therapies.

腺苷通路通常是指腺苷及其受体以及腺苷代谢过程涉及的多种酶和代谢产物。对腺苷通路调节免疫的研究可以追溯到约半个世纪以前^[1]^，近十余年来，其在抗肿瘤免疫方面的作用受到广泛关注。体内外试验发现，腺苷/腺苷受体可以抑制抗肿瘤免疫反应，尤其腺苷A2A受体（adenosine A2A receptor, A2AR）在免疫细胞上广泛表达且亲和力高，在肿瘤的免疫逃逸和现有的肿瘤治疗方案耐药中起到关键作用。因此，腺苷/A2AR通路阻断成为肿瘤免疫治疗的重要方向。在这篇综述中，我们将梳理阻断腺苷通路治疗肿瘤的理论机制，总结腺苷/A2AR通路阻断在肿瘤治疗中已有的研究结果，并对其在未来的应用前景进行展望。

## 1 肿瘤微环境中胞外腺苷水平升高

腺苷代谢在正常组织和肿瘤组织中广泛存在，在细胞内外都可代谢产生腺苷（图 1）。细胞外腺苷的产生主要有两条途径，典型途径是细胞外三磷酸腺苷（adenosine triphosphate, ATP）在外核苷酶CD39作用下去磷酸化生成一磷酸腺苷（adenosine monophosphate, AMP），AMP在外核苷酶CD73作用下生成腺苷^[2]^。除上述途径外，胞外烟酰胺腺嘌呤二核苷酸（nicotinamide adenine dinucleotide, NAD^+^）可在CD38和CD203a作用下生成AMP^[3]^，继而经CD73水解生成腺苷。腺苷在细胞外代谢极快，仅存在数秒的时间^[4]^，其去路包括作用于相应的受体调节代谢，或被腺苷脱氨酶（adenosine deaminase, ADA）降解生成肌苷^[5]^，或被位于细胞膜上的核苷转运体转运至胞内^[6]^。细胞内也可以直接生成腺苷，因为细胞内存在可溶性CD73，可将胞内AMP水解生成腺苷^[7]^。胞内腺苷可经由腺苷激酶（adenosine kinase, ADK）作用生成AMP，或被ADA水解，或被转运体转运至胞外^[8]^。胞外的ATP是潜在的趋化因子和炎症刺激因子^[9]^，而胞外腺苷总体而言起到免疫抑制作用，生理状态下ATP和腺苷在细胞外的水平很低，且两者处于动态平衡中，使免疫反应的强度控制在恰当水平。但在肿瘤微环境（tumor microenvironment, TME）中，由于低氧、炎症反应、营养匮乏、细胞毒性药物的使用等种种因素^[9-11]^，ATP经由通道释放、囊泡释放、或细胞坏死直接释放等多种方式到达胞外，导致细胞外ATP生成增加（图 1）。同时，TME中促进ATP向腺苷转化的各种酶的表达也是升高的。在TME中肿瘤细胞、免疫细胞、内皮细胞、成纤维细胞等都有表达CD39和/或CD73^[12, 13]^，肿瘤细胞还可以产生携带CD39和CD73的外分泌体^[14]^。在这些酶的作用下腺苷生成明显增多。除CD39和CD73外，CD38依赖的通路也是导致TME中腺苷水平升高的重要途径。由于肿瘤代谢变异，胞外NAD^+^浓度升高，同时Ⅰ型干扰素、全反式维甲酸、免疫检查点抑制剂等都可以上调CD38的表达^[15]^。同时，TME低氧环境下通过低氧诱导因子1（hypoxia-inducible factor 1, HIF1）途径，腺苷水解酶ADA的表达受到抑制^[16]^。综上，在TME中大量ATP被水解生成腺苷，胞外NAD^+^在CD38作用下最终代谢生成腺苷，且ADA表达降低，导致胞外腺苷水平升高。

**图 1:**
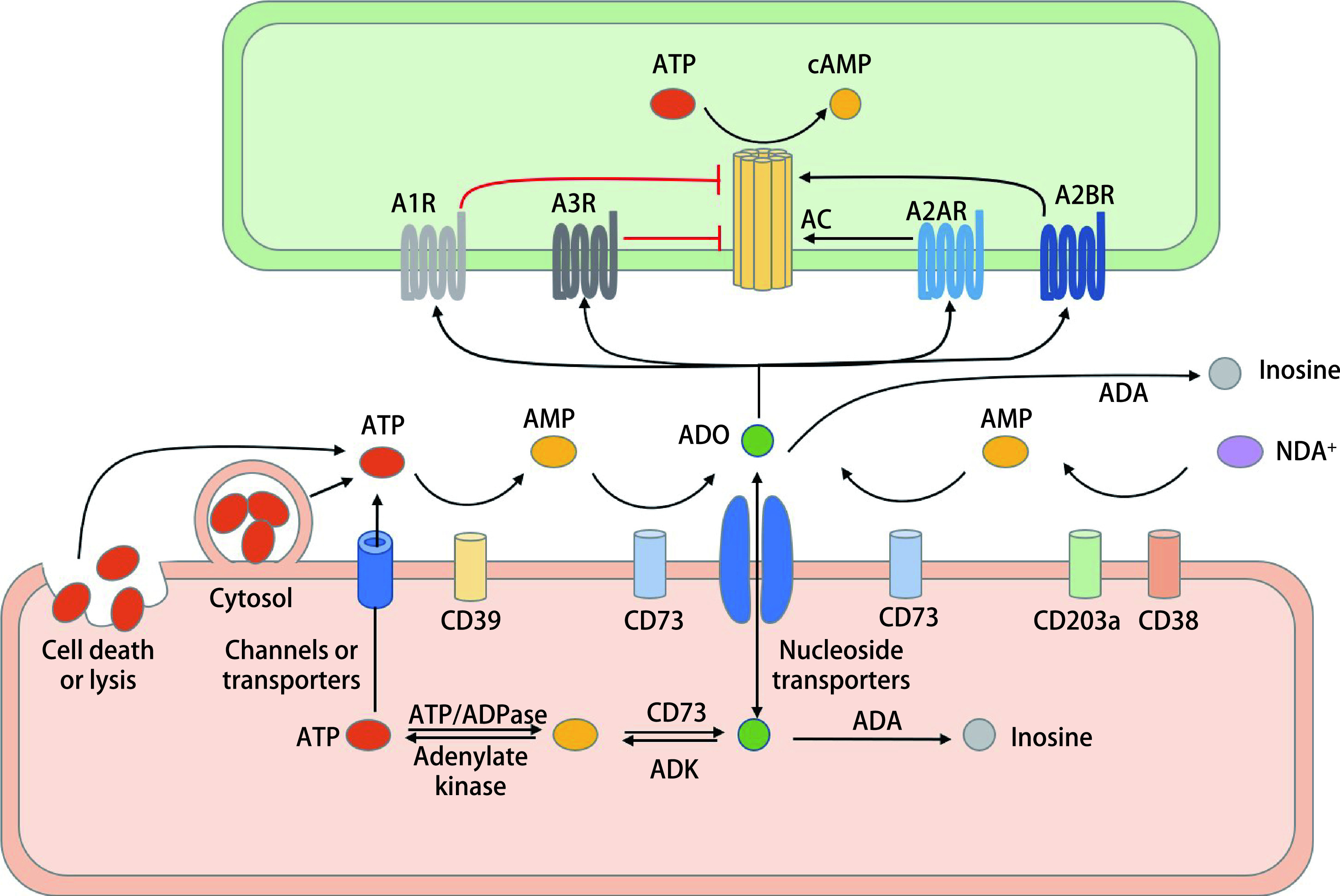
腺苷代谢通路。腺苷的前体ATP可由细胞裂解/死亡、胞吐、通道释放或转运体等多种方式释放到细胞外。ATP经CD39酶解生成AMP，AMP经CD73生成腺苷，这是腺苷生成的经典途径。此外，NAD^+^也可作为底物，在CD38和CD203a作用下生成AMP进而生成腺苷。细胞外腺苷可作用于四种受体（A1R、A2AR、A2BR、A3R）发挥作用，或被ADA代谢生成肌苷，或被核苷转运体转运至胞内。在细胞内，腺苷和AMP、AMP和ATP可在酶的作用下相互转化。在腺苷的四种受体中，A1R和A3R抑制AC，下调cAMP，而A2AR和A2BR激活AC，上调cAMP，cAMP在免疫调节中发挥重要作用。ATP：三磷酸腺苷；AMP：一磷酸腺苷；ADO：腺苷；NAD^+^：烟酰胺腺嘌呤二核苷酸；ADA：腺苷脱氨酶；ADK：腺苷激酶；AC: 腺苷酸环化酶；cAMP：环磷酸腺苷。 Adenosine metabolic pathway. ATP, the precursor of ADO, can be released into extracellular space by cell lysis/death, exocytosis, channels or transporters. Sequential hydrolysis of ATP to AMP by CD39 and AMP to ADO by CD73 is the canonical pathway to produce extracellular ADO. In addition, NAD^+^ can also act as a substrate to generate AMP and thus adenosine by CD38 and CD203a. Extracellular ADO can act on four receptors (A1R, A2AR, A2BR, and A3R), or be metabolized by ADA to generate inosine or transported into cells by nucleoside transporters. In the intracellular space, ADO and AMP, AMP and ATP can be converted to each other by enzymes. Among the four adenosine receptors, A1R and A3R inhibit AC and down-regulate cAMP while A2AR and A2BR activate AC and up-regulate cAMP, which play an important role in immune regulation. ATP: adenosine triphosphate; AMP: adenosine monophosphate; ADO: adenosine; NAD^+^: nicotinamide adenine dinucleotide; ADA: adenosine deaminase; ADK: adenosine kinase; AC: adenylate cyclase; cAMP: cyclic adenosine phosphate.

## 2 腺苷/A2AR通路激活导致肿瘤免疫逃逸

胞外腺苷可作用于4种嘌呤能G蛋白偶联受体，即A1受体（A1R）、A2AR、A2B受体（A2BR）和A3受体（A3R）^[17]^，这4种受体在多种细胞上都有表达，包括免疫细胞。其中A1R和A3R主要表达于中性粒细胞，调节中性粒细胞的趋化作用；A2AR在T细胞、自然杀伤T细胞、单核细胞、巨噬细胞、树突状细胞（dendritic cells, DCs）、自然杀伤（natural killer, NK）细胞广泛表达，起到抗炎和抑制免疫的作用；A2BR主要在巨噬细胞和DCs上表达，其激活能够促进白细胞介素-6（interleukin-6, IL-6）和血管内皮生长因子（vascular endothelial growth factor, VEGF）的释放^[18]^。但A2BR在4种受体中亲和力最低，在生理状态下极少被激活^[18]^。总之，A2AR在免疫细胞上分布最为广泛，亲和力相对较高，其在免疫细胞中的作用得到了较为全面和深入的研究。

腺苷/A2AR激活可活化下游的腺苷酸环化酶（adenylate cyclase, AC）从而上调环磷酸腺苷（cyclic adenosine monophosphate, cAMP）水平^[17]^，cAMP可通过蛋白激酶A（protein kinase A, PKA）影响免疫细胞和免疫过程。在效应T细胞中，A2AR信号引起的PKA激活具有多种抑制作用，包括：①通过阻断Zeta链相关蛋白激酶的激活来抑制近端T细胞受体信号^[19, 20]^；②抑制多种丝裂原活化蛋白激酶（mitogen-activated protein kinase, MAPK）的活性^[19]^；③抑制蛋白激酶C（protein kinase C, PKC）的活性，而PKC在效应细胞活化中起重要作用^[18]^；④引起cAMP应答元件结合蛋白（cAMP-responsive element-binding protein 1, CREB）活化，对核因子κB（nuclear factor-κB, NF-κB）和活化T细胞核因子（nuclear factor of activated T cells, NFAT）起抑制作用^[21]^；⑤抑制T细胞增殖^[18]^。A2AR激活还可抑制CD4^+^T细胞分泌白细胞介素2（interleukin-2, IL-2）并抑制共刺激受体CD28的表达^[22]^。在效应性CD8^+^T细胞中，腺苷/A2AR通路对细胞增殖、细胞因子产生及细胞杀伤作用均有抑制作用^[18]^。无论是效应T细胞还是调节T细胞（regulatory T cells, Treg），A2AR可增加程序性死亡受体1（programmed cell death protein 1, PD-1）、细胞毒性T淋巴细胞相关抗原4（cytotoxic T lymphocyte antigen 4, CTLA-4）、淋巴细胞活化基因3蛋白（lymphocyte activation gene 3 protein, LAG-3）、T细胞免疫球蛋白粘蛋白分子3（T cell immunoglobulin and mucin domain-containing protein 3, TIM-3）等免疫检查点的表达^[13, 18, 23]^，并促进CD4^+^T细胞向Treg细胞分化。最后，A2AR还可通过上调Foxp3表达来稳定Treg细胞^[24]^。

除T细胞以外，A2AR也对其他免疫细胞进行调节。A2AR激活可抑制NK细胞成熟^[25]^。在NK细胞中，A2AR激活在转录水平诱导Foxo-1表达升高，可能是通过此途径限制NK细胞成熟和功能^[26]^。在巨噬细胞中，A2AR激活使其分化为抑制免疫的M2样表型，产生白细胞介素20（interleukin-20, IL-20）、VEGF和精氨酸酶^[27, 28]^，利于肿瘤生长。在DCs中，A2AR信号抑制DCs成熟，诱导DCs分化为免疫抑制的表型，分泌白细胞介素10（interleukin-10, IL-10）、VEGF、转化生长因子β（transforming growth factor β, TGFβ）、精氨酸酶和免疫调节酶吲哚胺2, 3-双加氧酶（indoleamine 2, 3-dioxygenase, IDO）、环氧化酶（cyclooxygenase 2, COX2），抑制IL-2和CD80/CD86共刺激分子^[25]^。体外实验中，暴露于腺苷的DCs可让T细胞失去效应能力^[29]^。

如前所述，在TME中，由于低氧、营养不足及炎症状态等因素，ATP大量释放至胞外。ATP本身可作用于免疫细胞上的P2X7受体促进抗肿瘤免疫应答^[10]^。但TME中大量ATP转化生成腺苷，这一方面导致了胞外ATP水平降低，另一方面，腺苷通过A2AR等受体发挥免疫抑制作用，从而限制了炎症反应的强度和抗肿瘤免疫的力度。此外，肿瘤细胞本身也表达腺苷受体，胞外腺苷可直接作用于肿瘤细胞促进其生长和转移。例如，在胃癌模型中，A2AR信号作用于肿瘤细胞，激活PI3K-AKT-mTOR信号通路，促进肿瘤生长和转移^[30]^。综上所述，TME中腺苷水平升高和A2AR激活成为肿瘤免疫逃逸的重要途径。因此，对腺苷/A2AR通路的阻断有望增强抗肿瘤免疫应答，进而改善肿瘤患者的预后。

## 3 腺苷/A2AR通路阻断用于抗肿瘤治疗的临床前研究

早在十余年前，Sitkovsky团队^[31]^发现在*A2AR*基因敲除的小鼠中，通过CD8^+^T细胞依赖的途径，肿瘤的生长被完全抑制，而且特异性敲除T细胞的A2AR和A2BR可抑制肿瘤转移，延长生存。他们还在肉瘤模型和免疫原性差的LL-LCMV肿瘤模型中证明了药物阻断A2AR可以增强T细胞介导的抗肿瘤作用^[31]^。另有研究^[32]^表明，在小鼠乳腺癌和黑色素瘤模型中，A2AR阻断能够抑制肿瘤的转移能力。Powell团队^[33]^报告了在A2AR缺失小鼠中，EL4肿瘤细胞生长受到抑制，并进一步证明在*A2AR*基因敲除小鼠模型中应用抗PD-1治疗可增强抗肿瘤效应。随后的研究^[34-36]^也表明，在多种肿瘤模型中，抑制A2AR和阻断免疫检查点（例如PD-1、TIM-3、CTLA-4等）联合应用可有效增强免疫介导的肿瘤控制。A2AR阻断还可以与CD73阻断联用。A2AR和CD73的阻断都是针对腺苷通路介导的免疫抑制，乍看这种联用策略似乎多此一举。但胞外腺苷可经由多种代谢途径产生（图 1），单一阻断CD73并不能完全抑制腺苷生成。另一方面，A2AR不是介导免疫抑制的唯一受体，在腺苷浓度较高的肿瘤微环境中，亲和力低的A2BR可能发挥作用导致肿瘤免疫逃逸的发生。因此，同时阻断A2AR和CD73在理论上可以实现增效的抗肿瘤能力。临床前研究也证实了这种联用策略的有效性：在动物模型中，与单药相比，A2AR和CD73双阻断确实可以进一步控制肿瘤生长和转移^[37]^。总之，腺苷/A2AR信号通路阻断的抗肿瘤效应在临床前研究中得到广泛证据支持，推动了该策略向临床应用的转化。

## 4 腺苷/A2AR通路阻断在抗肿瘤治疗中的初步探索

目前已有多种用于肿瘤治疗的A2AR拮抗剂在Ⅰ期-Ⅱ期临床试验中，包括CPI-444（Ciforadenant）、MK-3814A（Preladenant）、AZD4635、AB928（Etrumadenant）、TT-10、NIR178（Taminadenant）、DZD2269、EOS100850（Inupadenant）、CS3005、PBF-999、INCB106385（表 1）。其中AB928和INCB106385为A2AR和A2BR双阻断剂。初步试验结果表明A2AR拮抗剂在肿瘤患者中耐受良好。研究^[38-40]^报道CPI-444单药或联合Atezolizumab [程序性死亡配体1（programmed cell death ligand 1, PD-L1）单抗] 常见治疗相关不良反应（treatment-related adverse events, TRAEs）有疲倦、恶心、皮肤瘙痒等，3级-4级不良反应少见。AZD4635同样安全性良好^[41]^。NIR178单药用于晚期NSCLC的研究中，常见TRAEs为恶心、疲倦、胸闷等；3级不良反应有肺炎（8%）和恶心（3%）^[42]^。AB928在治疗前列腺癌的I期/ⅠB期临床试验中，最常见TRAEs为1级疲倦和恶心，44例患者中有1例发生3级肾损伤^[43]^。EOS100850的常见不良反应有恶心、疲倦、呕吐等，没有观察到3级及以上TRAEs或剂量限制性毒性^[44]^。

**表 1:** A2AR拮抗剂临床试验概览 Clinical trials related to A2AR antagonists

Drugs	Company	ClinicalTrials.gov Number	Study phase	Combination	Cancer type	Results	Ref
CPI-444 (Ciforadenant)	Corvus	NCT02655822	Ⅰ/ⅠB	Monotherapy or combined with atezolizumab (anti-PD-L1)	RCC mCRPC	ORR 3.0% (1/33)(monotherapy, RCC) ORR 11.4% (4/35)(combination, RCC) ORR 6.4% (3/47)(mCRPC)	[38, 40]
		NCT04280328	ⅠB	Daratumumab (anti-CD38)	Relapsed or refractory multiple myeloma		
		NCT03337698	ⅠB/Ⅱ	Atezolizumab	NSCLC		
		NCT03454451	Ⅰ/ⅠB	CPI-006 (anti-CD73)	Advanced solid tumor		
MK-3814A (Preladenant)	Merck	NCT03099161	ⅠB/Ⅱ	Monotherapy or combine with pembrolizumab (anti-PD-1)	Advanced solid tumor		
AZD4635	AstraZeneca	NCT02740985	Ⅰ	Monotherapy or combined with Durvalumab (anti-PD-L1)，Oleclumab (anti-CD73)，chemotherapy	Advanced solid tumor	ORR 6.1% (2/33) (monotherapy) ORR 16.2% (6/37)(combine with Durvalumab)	[46]
		NCT04089553	Ⅱ	Durvalumab or Oleclumab	Prostate cancer		
		NCT03980821	Ⅰ	Monotherapy	Advanced solid tumor		
		NCT04495179	Ⅱ	Durvalumab±chemotherapy	mCRPC		
		NCT03381274	ⅠB/Ⅱ	Oleclumab	NSCLC (*EGFR* mutation)		
TT-10	Tarus Therapeutics	NCT04969315	Ⅰ/Ⅱ	Monotherapy	Advanced solid tumor		
AB928* (Etrumadenant)	Arcus Biosciences	NCT04892875	ⅠB	Zimberelimab (anti-PD-1), chemoradiotherapy	Locally advanced head and neck cancers		
		NCT03719326	Ⅰ/ⅠB	Chemotherapy±IPI-549 (PI3K-*γ* inhibitor)	TNBC, ovarian cancer		
		NCT03720678	Ⅰ/ⅠB	Chemotherapy	Gastrointestinal malignancies	ORR 9.1% (*n*=22)	[43]
		NCT04660812	ⅠB/Ⅱ	Zimberelimab+chemotherapy±Bevacizumab (anti-VEGF); Zimberelimab+AB680（CD73 inhibitor）	Metastatic colorectal cancer		
		NCT03629756	Ⅰ	Zimberelimab	Advanced solid tumor		
		NCT04262856	Ⅱ	Zimberelimab+ Domvanalimab (anti-TIGIT)	NSCLC (PD-L1 positive)		
		NCT03846310	Ⅰ/ⅠB	Chemotherapy, Pembrolizumab, Zimberelimab	NSCLC	ORR 42.9% (3/7)	[45]
		NCT04381832	ⅠB/Ⅱ	Zimberelimab, AB680, chemotherapy	mCRPC		
		NCT05024097	Ⅱ	Chemotherapy, radiotherapy, Zimberelimab	Rectal cancer		
		NCT05177770	Ⅱ	SRF617 (anti-CD39)+ Zimberelimab	mCRPC		
		NCT04791839	Ⅱ	Zimberelimab+Domvanalimab	NSCLC		
		NCT03821246	Ⅱ	Atezolizumab	Prostate cancer (neoadjuvant)		
		NCT03193190	ⅠB/Ⅱ	Atezolizumab+chemotherapy	Pancreatic adenocarcinoma		
NIR178/PBF-509 (Taminadenant)	Novartis	NCT03207867	Ⅱ	PDR001 (anti-PD-1)	Advanced solid tumor; Non-Hodgkin lymphoma		
		NCT04237649	Ⅰ/ⅠB	KAZ954	Advanced solid tumor		
		NCT03549000	Ⅰ/ⅠB	NZV930 (anti-CD73)±PDR001	Advanced solid tumor		
		NCT03742349	ⅠB	Spartalizumab (anti-PD-1)+ LAG525 (anti-LAG-3)	TNBC		
		NCT04895748	Ⅰ/ⅠB	DFF332 (Hif2α inhibitor)+ PDR001	Renal cancer		
		NCT02403193	Ⅰ/ⅠB	Monotherapy or combined with PDR001	NSCLC	ORR 8.3%（2/24）（monotherapy）	[42]
DZD2269	Dizal	NCT04634344	Ⅰ	Monotherapy	mCRPC		
EOS100850 (Inupadenant)	iTeos	NCT03873883	Ⅰ/ⅠB	Monotherapy or combine with Pembrolizumab/chemotherapy	Advanced solid tumor		
		NCT05117177	Ⅰ	Monotherapy	Advanced solid tumor		
		NCT05060432	Ⅰ/Ⅱ	EOS-448 (anti-TIGIT)	Advanced solid tumor		
CS3005	CStone	NCT04233060	Ⅰ	Monotherapy	Advanced solid tumor		
PBF-999	Palobiofarma	NCT03786484	Ⅰ/ⅠB	Monotherapy	Advanced solid tumor		
INCB106385*	Incyte	NCT04580485	Ⅰ	Monotherapy or combine with INCMGA00012 (anti-PD-1)	Advanced solid tumor		
		NCT04989387	Ⅰ	INCA00186 (anti-CD73)±Retifanlimab (anti-PD-1)	Advanced solid tumor		
*: A2AR & A2BR antagonist. RCC: renal cell cancer; mCRPC: metastatic castration resistant prostate cancer; NSCLC: non-small cell lung cancer; TNBC: triple-negative breast cancer; TIGIT: T cell immunoreceptor with Ig and ITIM domains; ORR: objective response rate; PD-1: programmed cell death protein 1; PD-L1: programmed cell death ligand 1.							

A2AR拮抗剂的安全性已经在临床试验初步结果中得到证明，但在肿瘤治疗疗效方面，A2AR拮抗剂单药使用疗效有限，与其他治疗方式联合应用似乎效果更优（表 1）。A2AR拮抗剂的联用策略多种多样，当前多项临床试验中，A2AR拮抗剂可与放化疗、PD-1/PD-L1单抗、CD73单抗、靶向药物等联用，各联用策略的优劣有待研究数据的进一步发表。值得注意的是，A2AR拮抗剂有可能逆转既往免疫治疗耐药的情况。CPI-444单药或联合Atezolizumab治疗复发性肾细胞癌的临床试验（NCT02655822）纳入的是前线经PD-1/PD-L1单抗治疗后进展的患者，初步结果报告CPI-444单药治疗组的33例患者中有1例部分缓解（partial response, PR），联合治疗组35例患者中有4例PR^[38]^。非小细胞肺癌患者应用AB928联合化疗/免疫治疗的试验中（NCT03846310），7例患者中有3例确认PR，其中包括1例前线Ipilimumab（CTLA-4单抗）联合Nivolumab（PD-1单抗）治疗后进展的患者^[45]^。虽然初步结果的样本量和有效患者的数目较少，但A2AR拮抗剂为改善免疫检查点抑制剂耐药带来了希望。

## 5 腺苷通路相关生物标志物

现有的临床前及临床研究已证实了A2AR拮抗剂的安全性和有效性，但在临床试验中其疗效似乎低于预期。因此，仍需探索相应的生物标志物来识别最可能获益的人群。例如筛选出TME中腺苷水平较高的患者，有针对性地加用A2AR拮抗剂从而获得最佳疗效。目前而言难以直接对腺苷水平进行测定，因为腺苷代谢快，在血浆中其半衰期仅10 s左右^[4]^，因此无法通过直接检测腺苷来作为生物标志物。腺苷相关的基因表达谱与肿瘤中腺苷水平存在一定的相关性，可能作为潜在的检测指标。有研究^[38]^发现与髓系细胞生物学和炎症相关的一组基因表达情况与腺苷水平呈正相关，并最终锁定了8个基因（*CXCL1*、*CXCL2*、*CXCL3*、C*XCL5*、*CXCL6*、*CXCL8*、*PTGS2*和*IL-1β*）作为“腺苷标签”（adenosine signature, AdenoSig）。在30例应用CPI-444单药或联合Atezolizumab治疗的肾细胞癌患者中，AdenoSig高表达与更长的无进展生存期相关^[38]^。另有一些其他基因系列提示可指导肿瘤免疫治疗的预后^[47]^，但目前尚无统一的结论。

CD39和CD73或许也可作相关的生物标志物。CD39既在浸润的免疫细胞上表达，也在肺癌、黑色素瘤、胰腺癌、淋巴瘤等多种肿瘤细胞上表达^[48-50]^。研究^[51-53]^发现CD39高表达与不良预后相关。同CD39一样，CD73也在肿瘤细胞和浸润的免疫细胞上表达。在乳腺癌、肺癌、卵巢癌、肾癌、胃癌、前列腺癌、膀胱癌、宫颈癌、黑色素瘤、头颈部肿瘤等多种肿瘤类型中均发现TME中CD73的表达水平与预后相关^[54]^。多数研究一致报道CD73高表达提示肿瘤预后不良，这与腺苷的免疫抑制作用相符合。然而也有部分研究^[55-58]^报道CD73高表达提示更好的临床结局，尤其是在疾病早期，这些研究涉及尿路上皮癌、子宫内膜癌及乳腺癌。一种可能的解释是CD73介导生成的腺苷可作为血管内皮的屏障从而抑制转移的发生^[54]^。但CD39和CD73能否作为预测A2AR拮抗剂疗效的生物标志物，目前尚无充分研究证据。

## 6 前景展望

随着我们对TME的认识越来越深入，肿瘤免疫治疗的应用将越来越广泛和细化。在未来的几年里，阻断腺苷途径以及其他潜在分子途径的药物可能单独使用或与传统疗法联合使用，将成为癌症治疗方法的重要组成部分。如何发掘生物标志物预测治疗效果、筛选患者人群，如何实现A2AR拮抗剂的最佳联用方式，仍需要进行更多探索工作，以期实现最优的诊疗策略，进一步改善癌症患者的临床结局。
